# Effect of Different Morphology of Titanium Surface on the Bone Healing in Defects Filled Only with Blood Clot: A New Animal Study Design

**DOI:** 10.1155/2018/4265474

**Published:** 2018-08-08

**Authors:** Sergio Alexandre Gehrke, Berenice Anina Dedavid, Jaime Sardá Aramburú, Letícia Pérez-Díaz, José Luis Calvo Guirado, Patrícia Mazon Canales, Piedad N. De Aza

**Affiliations:** ^1^Biotecnos Research Center, Montevideo, Uruguay; ^2^University Catholica San Antonio de Murcia (UCAM), Murcia, Spain; ^3^Instituto de Bioingenieria, Universidad Miguel Hernandez, Elche (Alicante), Spain; ^4^Department of Materials Engineering, Pontificial Catholic University of Rio Grande do Sul, Porto Alegre, Brazil; ^5^Biotecnos Research Center, Santa Maria, Brazil; ^6^Veterinary Department of Itapiranga Faculty, Itapiranga, Brazil; ^7^Laboratorio de Interacciones Moleculares, Facultad de Ciencias, Universidad de la Republica, Montevideo, Uruguay; ^8^International Dentistry Research Cathedra, Faculty of Medicine & Dentistry, San Antonio Catholic University of Murcia (UCAM), Murcia, Spain; ^9^Departamento de Ciencia de Materiales, Optical y Tecnologia Electrónica, Universidad Miguel Hernandez, Elche (Alicante), Spain

## Abstract

**Background:**

The objective of the present histologic animal study was to analyze whether roughness of the titanium surface can influence and/or stimulate the bone growth in defects filled with the blood using a rabbit tibia model.

**Materials and Methods:**

Forty sets (implant and abutment), dental implant (3.5 mm in diameter and 7 mm in length) plus healing abutment (2.5 mm in diameter), were inserted in the tibiae of 10 rabbits. Moreover, twenty titanium discs were prepared. The abutment and discs were treated by 4 different methods and divided into 4 groups: (group A) machined abutments (smooth); (group B) double acid etching treatment; (group C) treatment with blasting with particles of aluminum oxide blasted plus acid conditioning; (group D) treatment with thorough blasting with particles of titanium oxide plus acid conditioning. The discs were used to characterize the surfaces by a profilometer and scanning electronic microscopy.

**Results:**

After 8 weeks, the new bone formation around the sets of the samples was analyzed qualitatively and quantitatively in relation to bone height from the base of the implant and presence of osteocytes. Group C (1.50±0.20 mm) and group D (1.62±0.18 mm) showed bone growth on the abutment with higher values compared to group A (0.94±0.30 mm) and group B (1.19±0.23 mm), with significant difference between the groups (P < 0.05). In addition, osteocyte presence was higher in groups with surface treatment related to machined (P < 0.05).

**Conclusions:**

Within the limitations of the present study, it was possible to observe that there is a direct relationship between the roughness present on the titanium surface and the stimulus for bone formation, since the presence of larger amounts of osteocytes on SLA surfaces evidenced this fact. Furthermore, the increased formation of bone tissue in height demonstrates that there is an important difference between the physical and chemical methods used for surface treatment.

## 1. Introduction

Studies have demonstrated different superior survival rates of dental implants in the anterior mandible area (higher bone density) compared to the posterior maxilla (lower bone density) [1, 2]. Specific areas where there is a lower density of bone tissue that requires implants, such as the posterior maxilla region, where the predictability of osseointegration is lower, have been the subject of numerous researches in the sense of seeking new macro and micro structural drawings of the implant for increasing the predictability and the possibility of applying loads as early as possible [3–5]. Among the modifications in the sense of improving tissue response, surface treatment of implants has received more attention and is one of the most researched topics. Since the first portion of an implant to interact with the patient's tissues after implantation is the surface of the implant where direct contact occurs with the blood and consequently with its cellular components and growth factors, its morphological structure (roughness pattern) and/or physical-chemical characteristics are widely analyzed [6, 7]. The first step for the bone healing on the titanium implant (osseointegration) is forming a blood clot at the surface. The initial contact of blood with biomaterials and subsequent recruitment of inflammatory and marrow-derived stromal cells is among the first phases of bone regeneration [8]. Other authors related that they believe that the early blood cell/implant interactions may play a key role in the osteoconduction stage of peri-implant bone healing in response to micro-roughened implants [9].

Several studies have demonstrated that the surface roughness in titanium in comparison to smooth surfaces presents a result of osseointegration better after its placement in function supporting the masticatory loads [10–14]. Although it has been established that bone/implant contact can be accelerated by surfaces with moderate roughness when compared to smooth surfaces [15], recent studies have shown that the physical-chemical composition at nanometer scale can positively alter the cellular response and, consequently, accelerate the osseointegration process [16–18]. This morphology in nanometric parameters apparently allows a pattern of cellular activity and protein absorption easier. Moreover, most of the cellular components responsible and/or involved in the healing process of the bone tissue have a nanometric pattern [19]. All of these observations at the different scales (micro- or nanometric) on the relationship between the surface morphology of the implants and the cellular reactions increase the evidence that the physical-chemical modifications of the surface can alter the cellular activity and response during the healing process of the implants of tissues in contact with titanium treated [11, 13, 20].

The characterization of the surface of the materials is vital to know the structure and the biologic reaction. The cellular activities (adhesion and growth) on a surface is influenced directly by its morphological characteristics and its chemical composition [21]. Then, the purpose of this histological animal study was to analyze the effect of titanium surface with different roughness patterns on bone tissue formation in small defects (as a healing chamber) filled only with blood clot in the tibia of rabbits.

## 2. Materials and Method

### 2.1. Materials and Groups Presentation

**Figure 1:**
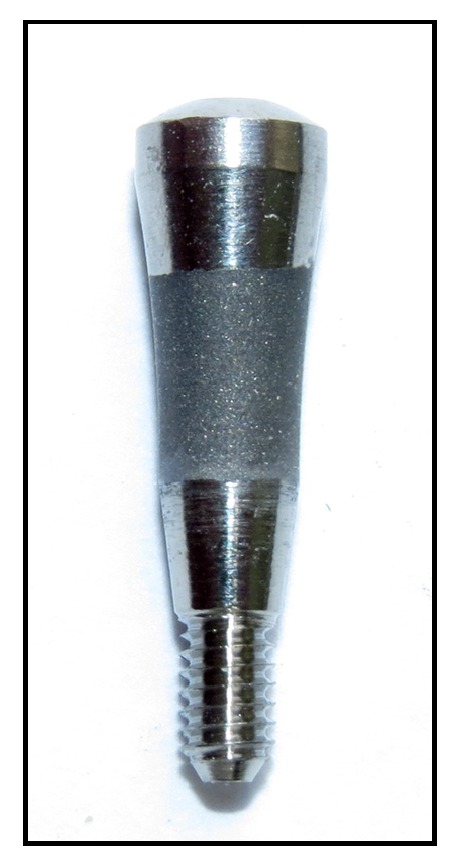
Image of the healing abutment used in this study, which received the surface treatment in the transmucosal portion.

Forty healing abutments, fabricated in commercially pure titanium (grade IV), with a 2.5 mm diameter and 4.5 mm transmucosal height and 20 titanium discs measuring 6 mm in diameter and 2 mm in length were prepared (Figure 1).

Alterations in the surface were obtained using 4 different treatments generating the groups: machined surface (Group A); surface conditioned by double acid etching using hydrofluoric acid (HF), following of sulfuric acid (H_2_SO_4_) solutions (Group B); surface blasted with aluminum oxide (Al_2_O_3_) microparticles (100 *μ*m) and passivated with nitric acid (HNO_3_) solution (Group C); surface blasted with titanium oxide (TiO2) particles (50-100 *μ*m) and passivated with maleic acid (HO_2_CCH_2_CHOHCO_2_H). Ten abutments and 5 discs were used in each group. All the samples used in the present study received the same care and treatments applied and required for the final commercialization of implantable products.

For the present study were used 40 Morse taper dental implants with the surface treatment equal as described for the treatment applied in the abutment of group D. The dimensions of the implants were 3.5 mm in diameter and 7 mm in length. All implantable materials used were produced by Implacil DeBortoli (São Paulo, Brazil).

### 2.2. Morphological Characterization of the Samples

The 5 discs of each group were characterized in scanning electron microscopy (Philips XL30, Eindhoven, The Netherlands) at ×1,000 to record a series of images based on secondary electrons (SEs) and submitted to the optical laser profilometer (Mahr GmbH, Gottingen, Germany) to evaluate the roughness of the surface of the sample of each group, measuring the high variation of the valleys (Z), the absolute values of all profile points (Ra), the root-mean-square of the values of all points (Rq), and the value of the absolute heights of the five highest peaks and the depths of the five deepest valleys (Rz).

### 2.3. Animal Surgery and Care

**Figure 2:**
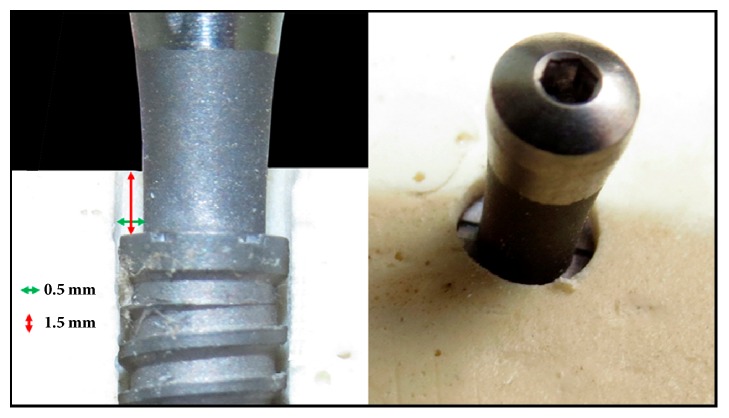
Schematic images showed the difference between the implant diameter and abutment diameter generating a bone gap of 0.5 × 1.5mm around of all sets.

For the present in vivo analysis, ten rabbits (*Oryctolagus cuniculus*) that weigh 4 ± 0.5 kg were included. The protocol was evaluated and approved in the ethical committee of the Itapiranga Faculty, Itapiranga, Santa Catarina, Brazil (#004-09-2015). The anesthesia of the animals was performed by intramuscular (IM) injection of ketamine (35 mg/kg; Agener Pharmaceutical, Brazil). Subsequently, a muscle relaxant (Rompum 5 mg / kg, Bayer, Brazil) and a tranquilizer (Acepran 0.75 mg / kg, Univet, Brazil) were intramuscularly injected. To increase the control of pain and reduce bleeding, local anesthetic (3% Prilocaine-Felypressin, Astra, São Paulo, Brazil) was administered in the area corresponding to the surgical sites. Then, an incision was made by planes (external and internal) to access the bone tissue of each tibia. The bone bed to install the set (implant and abutment) was performed using a drill sequence recommended for this implant model under copious saline irrigation. Each animal received 1 set (implant + abutment) from each group, 2 implants per tibia (4 per animal). The position was defined by randomization (www.randomization.com) prior to the surgeries. All implants were installed 1.5 mm below the level of the cortical bone, being stabilized in the inferior cortical portion, and subsequently, the abutment was positioned. So, the difference between the implant diameter and abutment diameter generated a bone gap of 0.5 × 1.5mm around of all sets, in accordance with the scheme of Figure 2.

The rabbit represents a test system commonly used in orthopedics, and the tibia was selected as the implant site because of the simplicity of the surgical access [22]. During the implants placement, the implant initial stability was controlled by surgeon experience (SAG). The suture was performed by planes (internal and external) with catgut and nylon sutures, respectively. Postoperatively, 600,000 IU Benzetacil was administered by IM injection (single dose). Postsurgically, each animal was placed in individual cages with 12-hour cycles of light, temperature controlled in ~21°C, and the diet ad libitum. During the postoperative period, no complications were observed with any of the animals included in the present study. The euthanasia of the animals was performed by ketamine (2 ml) and xylazine (1 ml) overdose 8 weeks after implantation. Osseointegration of the implants is considered to be completed after the 8-week period in this animal model [23]. After removing all tibias of the animals, these were immediately immersed in formaldehyde-based fixative.

### 2.4. Histologic Procedures

**Figure 3:**
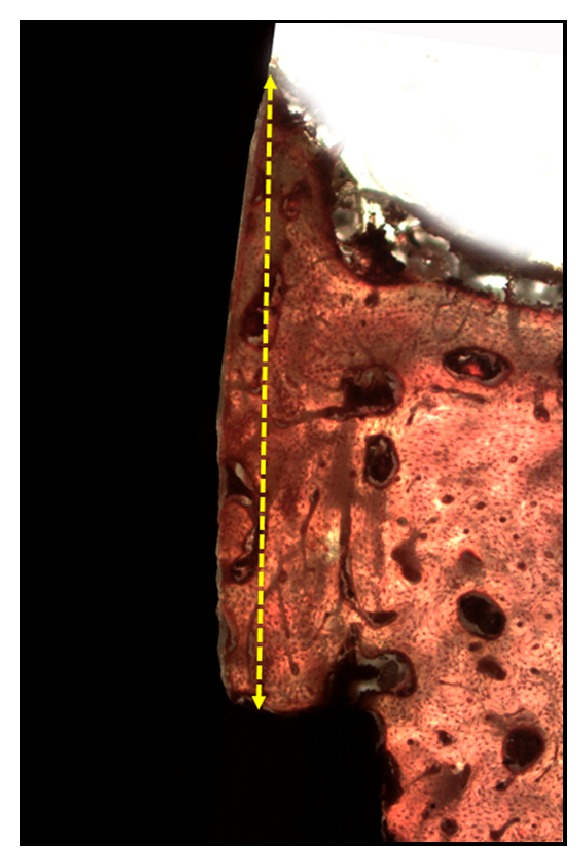
Image showed the height bone growth was measured with respect to the implant platform at the bone contact with the healing abutment.

**Figure 4:**
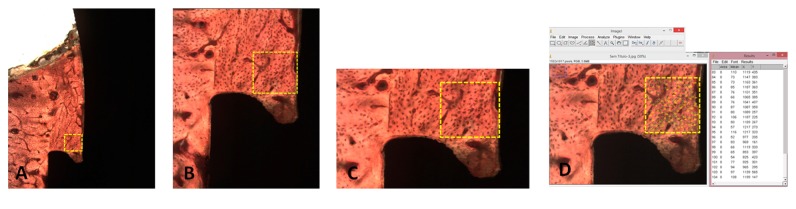
Scheme of the area predetermined (0.25mm2) to count the osteocytes. In (D), the osteocytes are counted in the ImageJ program.

The specimens collected from the animals (implant + abutment) integrated into the tibia bone were fixed (10% formaldehyde) for 10 days, after which the pieces were cut in small blocks and immersed in different concentrations of ethanol (60%, 70%, 80%, and 99%) for 24-56 h for dehydration [24]. Then, these dehydrated small blocks were embedded in Technovit 7200 VLC resin (Kultzer & Co., Wehrhein, Germany) and, after the polymerization, were sectioned using a metallographic cutter (Isomet 1000; Buehler, Germany). The cut slices were abraded in a bench polisher (Metaserv 3000; Buehler, Germany) using progressive (180, 220, 360, 600, and 1200 mesh) abrasive papers to achieve a thickness of ~30 *μ*m. After completion of the preparation of the slides, they were taken by light microscopy (Nikon E200, Tokyo, Japan) to analyze and obtain the images. The new bone formation in height, taking into account the implant platform to the highest point of the bone tissue in contact with the healing abutment, is shown in Figure 3. The count of osteocytes was made in a predetermined area of 0.25mm^2^ conforming with Figure 4, where the more internal area of the bone chamber is used, i.e., between the base of the implant platform and the abutment wall. All measurements and count were performed using* Image Tool *software, version* 5.02 *for* Microsoft Windows™. *The measurements were performed by two authors (SAG and MPGR), and a mean of these measured values was elaborated and considered for evaluation. However, the measurements were redone by the examiners every time the measured values were discrepant. The cell count was performed in 2 sides of each sample and a mean was made for each implant.

### 2.5. Statistical Analyses

The data measured for each group were analyzed longitudinally using the one-way analysis of variance (ANOVA) for repeated measures. The comparative analysis between groups was performed using the paired* t-*test. These statistical comparisons were made through the software* GraphPad Prism 5.0 *for* Windows* (GraphPad Software Inc., San Diego, CA, USA). In all analyses, significant differences were considered when p <0.05.

## 3. Results

All sets (implant + abutment) showed a strong stability after 8 weeks, showing that they are osseointegrated. No signs of infection were detected during the 8 weeks at any surgical site.

### 3.1. Disks Analysis of the Surface Morphology

**Figure 5:**
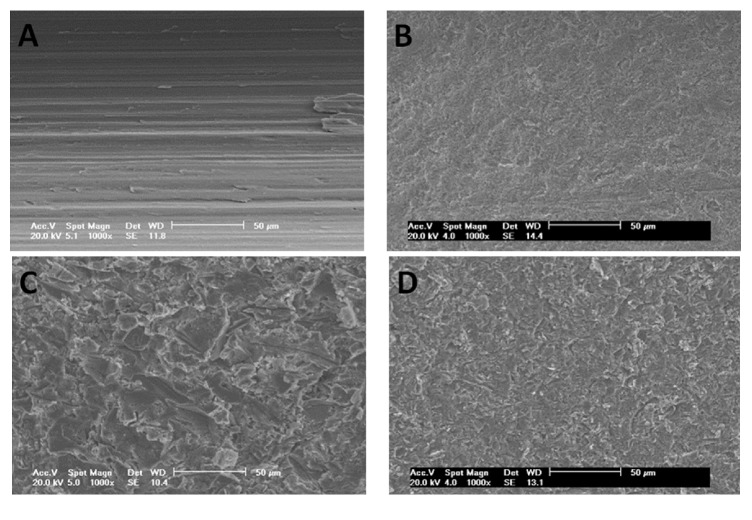
SEM of the surfaces after the treatment on the titanium discs of group A, group B, group C, and group D.

The observation of the images obtained in SEM showed different configuration in groups C and D, which presented more roughness than groups A and B (Figure 5).

The mean and standard deviation data of roughness parameters Z, Rq, Ra, and Rz are presented in Table 1 for each group.

### 3.2. Histologic Observations and Histomorphometry

**Figure 6:**
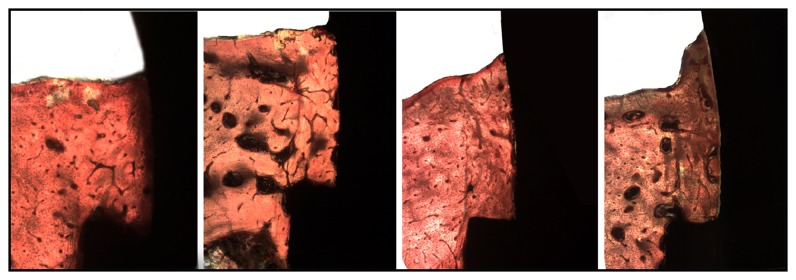
Histological images 8 weeks after healing of group A, group B, group C, and group D, respectively.

Complete bone neoformation was observed around all sets (implants + abutments) of all groups. The characteristics of the growth of bone tissue around the abutments were similar between the groups, with qualitative difference in the samples of group A (Figure 6).

**Figure 7:**
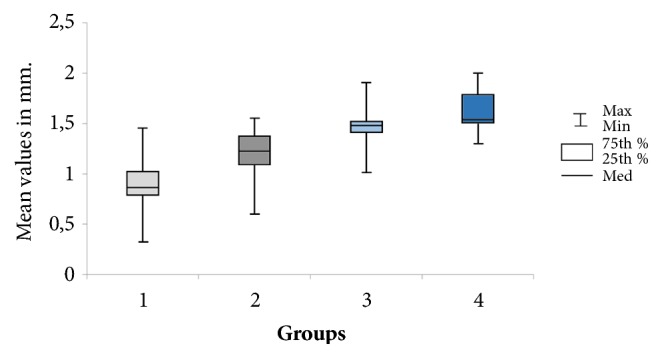
Box plots graph of the bone height measured in accordance with the scheme of Figure 3 in each group.

The mean of the bone measured for each group and the standard deviation were 0.94 ± 0.30 mm (range: 0.55–1.80 mm; length variation (ΔL) = 1.25 mm) for group A; 1.19 ± 0.23 mm (range: 0.60–1.55 mm; ΔL = 0.95 mm) for group B; 1.50 ± 0.20 mm (range: 1.01–1.90 mm; ΔL = 0.89 mm) for group C; and 1.62 ± 0.18 mm (range: 1.30–1.99 mm; ΔL = 0.69 mm) for group D. The measured values for each group are presented comparatively in the graph of Figure 7.

Significant difference by applying the ANOVA test was observed among data measured for the 4 groups studied (p< 0.001). In all cases, Fcal = 34.2104 was greater than F-crit = 2.7249, with significance set at p = 4.24^−14^.

**Figure 8:**
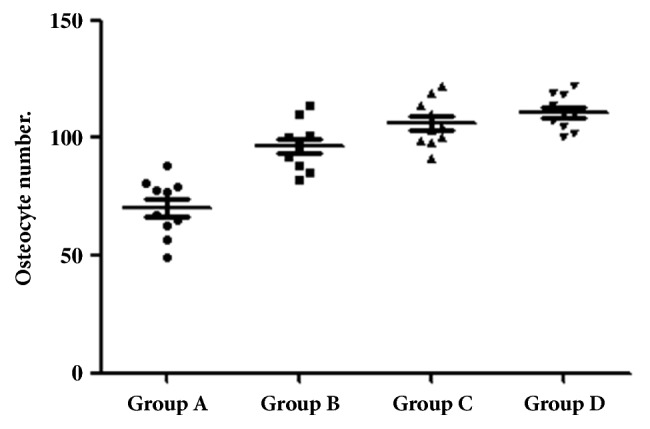
Point graph with the mean and standard deviation of the osteocytes count in accordance with the scheme of Figure 4 for each group.

The osteocytes counts in the predetermined area for each group were 70.4 ± 12.2 for group A, 96.5 ± 10.4 for group B, 106.1 ± 9.9 for group C, and 110.7 ± 7.4 for group D. These data are presented comparatively among the 4 groups in the graph of Figure 8, where a significant difference was observed using a one-way ANOVA test (p = 0.009).  Table 2 showed the statistical results of the comparison between each of the 2 groups.

## 4. Discussion

The objective of the present histologic animal study was to analyze whether roughness of the titanium surface can influence and/or stimulate the bone growth in defects filled with the blood using a rabbit tibia model. The present study developed the hypothesis presented in vitro by* Yang et al*. [8], which showed that the roughness presented on the titanium surface influences positively the formation of new bone tissue in the presence of blood (clot), which induces cellular settlement and, consequently, stimulates tissue healing. After previous study to determine if the surface treatment of the abutments could increase influence, the response of peri-implant tissues [14], using the platform reduction concept (switching platform) shown in Morse taper implants, glimpses the possibility of creating a new model to evaluate in vivo different surface only in the presence of the blood clot, simulating the healing chamber recently proposed for dental implants [25–27]. The calls healing chambers (empty space between the implant and the bone tissue) are immediately filled with blood clot that evolves towards the osteogenic tissue subsequent ossification through a pathway similar to intramembranous ossification [28], as was observed in the histological findings of the present study [28].

Various physical and chemical modifications on implant surfaces have been developed and presented commercially by the various manufacturers of these materials [29]. However, regarding the ideal condition for the growth of bone tissue on the titanium surface, there is no consensus so far. However, the surface morphology of the implants, which has been studied and worked at the micro- and nanometric level, can positively alter the activity and response of the peri-implant tissues. Currently, the mechanisms involved in the processes of bone healing, when in contact with a surface (treated or not), are not fully discovered and/or detailed. The modifications performed on the surface altering the physical and chemical characteristics directly affect the cellular activities (adhesion, proliferation, division, cell matrix production, among others) involved in the process of bone healing at the interface with the implant [30–32]. Currently, a large part of the implant-producing industry uses sandblasting and acid conditioning (SLA) for surface treatment of implants, in which sandblasting is performed by an abrasive particle (e.g., aluminum oxide, titanium oxide) with predetermined size, and then acid etching with a solution prepared at controlled temperature and time, as it is heavily backed and documented in the world literature [33, 34]. This type of treatment involving two processes (sandblasting and acid etching) is characterized by producing a surface with moderate roughness (2-4 *μ*m) by the acid attack on a rougher surface produced by blasting. Even if this surface treatment model is well documented, during the blasting process, when made with aluminum oxide, debris from this material can become impregnated at the surface [35] and can cause complications for long-term osseointegration [11, 20, 36]. In view of this possibility of surface contamination, other abrasive agents have been proposed and studied, with biocompatibility characteristics, such as bioactive calcium phosphate ceramics [37] and titanium oxide [38, 39]. For such materials cited as an alternative to aluminum oxide blasting, calcium phosphate is a resorbable material and, titanium oxide, has the same properties as the titanium implant, which demonstrated an excellent biologic response. In the present study were tested surfaces prepared using chemical (acids) and physical (blasting) processes, and qualitative and quantitative important differences were found. Even if group C presented higher roughness values than group D (Table 1), it is possible that the superior result found in group D of height measuring and cell count is related to the chemical composition resulting from the blasting medium used.

Some studies associate the high torque for the primary stabilization of the implant, at the moment of its insertion in the bone tissue, to the success in obtaining the osseointegration [39, 40]. However, this high torque is obtained at the expense of the structure presented by the cortical bone, which has a lower cellular response activity due to the low vascularization. Based on this concept, our experimental study aimed to evaluate the healing of cortical bone tissue, which is considered to be a slow response power to trauma, in a condition where there is no compression of the implant to this tissue and only contact with the blood present on the surface of the implant (Figure 4); however, to obtain the initial stability of the implants, and in this case to allow osseointegration, milling and fixation were achieved in the contralateral cortical of the tibia. In this sense, it has been previously demonstrated by other authors that bone defects of approximately 1 mm between the implant and the bone tissue can obtain the formation of a new bone in that place without the filling of this space by some type of material [41]. The results of the present study showed that concept (healing chambers) can be improved in the bone tissue healing around the titanium surface.

*Shiu *et al. (2014) [42] reported in the study that the clot formation mode determines its behavior in the neoformation of the bone tissue, and all the chemical modifications of the surface of the materials that make contact like the blood (during clot formation) can affect these processes in some way (positive or negative). In this way, new directions can be seen where the change of the clot is in contact with the surface of the materials, which could be another factor to be controlled and used positively in the search for the greater predictability possible for the biomaterials implanted in the human body. In this study, the data collected showed a significant difference between the groups proposed with different surface roughness, confirming the hypothesis suggested by* Parke & Davis *(2000) [9], where the initial contact between blood cells and the surface of the implant plays a fundamental stage in osteoconduction of the peri-implant repair bone tissue when there is roughness on the titanium surface.

Osteocytes are considered highly active multifunctional cells, being able to conduct virtually a large part of the metabolic processes of the skeleton, from the modeling and remodeling of the bone tissue as well as the substitution of minerals of the bone surfaces [43]. During development, proliferating cells can produce a predictable amount of extracellular matrix per cell and thereby control the mass of tissue formation directly by control of cell number [40]. Bonewald in 2006 [44] related that the osteocytes are the gatekeepers of bone formation and remodeling. Other studies related that cells are able to translate mechanical shear strain into biochemical signals that can communicate with other cells to affect remodeling [45–48]. In this sense, our quantitative metric of bone healing of the 4 different titanium surfaces was the cellular number, specifically, osteocyte count. The results showed different cells number in the proposed groups (A<B<C<D); however, significant statistical difference among the groups was observed in the comparison of group A versus the other 3 groups (B-C).

The limitations of the present study are mainly related to the amount of samples tested for each surface model and the conditions of the place where they were implanted, which are completely different from the conditions of use in humans (oral cavity). Then, other studies are fundamental to evaluate the effects after the application of functional loads on the implants and/or materials where the bone tissue was newly formed from the clot only, different from where the bone tissue already had its structure formed and it passes through a remodeling only. In addition, this in vivo study model to verify the potential of bone healing stimulation by the different surface can be very helpful. In this way, to examine the inflammatory responses, for example, several proinflammatory mediators including cytokines and prostanoid mediators should be examined and compared using different titanium surfaces. Furthermore, the osteocytes could be analyzed with several antibodies to distinguish the bone resorption status.

## 5. Conclusions

Within the limitations of the present study, it was possible to observe that there is a direct relationship between the roughness present on the titanium surface and the stimulus for bone formation, since the presence of larger amounts of osteocytes on SLA surfaces evidenced this fact. Furthermore, the increased formation of bone tissue in height demonstrates that there is an important difference between the physical and chemical methods used for surface treatment.

## Figures and Tables

**Table 1:** Mean and standard deviation of the surface groups profilometry (in *μ*m).

Rugosity parameters	Z	Rq	Ra	Rz
**Group A**	0.91 ± 0.11	0.15 ± 0.01	0.12 ± 0.02	0.94 ± 0.10
**Group B**	1.96 ± 0.13	0.42 ± 0.06	0.29 ± 0.03	1.26 ± 0.09
**Group C**	3.84 ± 1.18	0.93 ± 0.07	0.70 ± 0.05	3.12 ± 0.91
**Group D**	2.93 ± 1.02	0.82 ± 0.19	0.56 ± 0.10	2.59 ± 0.89

Z indicates longest distance recorded between the peak and the valley, high variation of the valleys; Ra, arithmetic average of the absolute values of all profile points; Rq, the root-mean square of the values of all points; Rz, the average value of the absolute heights of the 5 highest peaks and the depths of the 5 deepest valleys.

**Table 2:** Statistical *t*-test comparing the data among the 4 proposed groups.

	**Group A**	**Group B**	**Group C**	**Group D**
**Group A**	- - -	0.0005*∗*	< 0.0001*∗*	< 0.0001*∗*
**Group B**	0.0005*∗*	- - -	0.0588	0.0051
**Group C**	< 0.0001*∗*	0.0588	- - -	0.1202
**Group D**	< 0.0001*∗*	0.0051	0.1202	- - -

*∗*Statistical significative difference with p < 0.05.

## Data Availability

The images and tables data used to support the findings of this study are included within the article.
